# Effect of Cellulose Nanocrystals and Bacterial Cellulose on Disintegrability in Composting Conditions of Plasticized PHB Nanocomposites

**DOI:** 10.3390/polym9110561

**Published:** 2017-10-28

**Authors:** Irene Teresita Seoane, Liliana Beatriz Manfredi, Viviana Paola Cyras, Luigi Torre, Elena Fortunati, Debora Puglia

**Affiliations:** 1 Instituto de Investigaciones en Ciencia y Tecnología de Materiales (INTEMA), UNMdP, CONICET, Facultad de Ingeniería, Av. Juan B Justo 4302, B7608FDQ Mar del Plata, Argentina; itseoane@fi.mdp.edu.ar (I.T.S.); lbmanfre@fi.mdp.edu.ar (L.B.M.); vpcyras@fi.mdp.edu.ar (V.P.C.); 2 Civil and Environmental Engineering Department, University of Perugia, UdR INSTM, Strada di Pentima 4, 05100 Terni, Italy; luigi.torre@unipg.it (L.T.); elena.fortunati@unipg.it (E.F.)

**Keywords:** poly(3-hydroxybutyrate), cellulose nanocrystals, bacterial cellulose, plasticizers, biodegradable nanocomposites, disintegrability, compost

## Abstract

Poly(hydroxybutyrate) (PHB)-based films, reinforced with bacterial cellulose (BC) or cellulose nanocrystals (CNC) and plasticized using a molecular (tributyrin) or a polymeric plasticizer (poly(adipate diethylene)), were produced by solvent casting. Their morphological, thermal, wettability, and chemical properties were investigated. Furthermore, the effect of adding both plasticizers (20 wt % respect to the PHB content) and biobased selected nanofillers added at different contents (2 and 4 wt %) on disintegrability in composting conditions was studied. Results of contact angle measurements and calorimetric analysis validated the observed behavior during composting experiments, indicating how CNC aggregation, due to the hydrophilic nature of the filler, slows down the degradation rate but accelerates it in case of increasing content. In contrast, nanocomposites with BC presented an evolution in composting similar to neat PHB, possibly due to the lower hydrophilic character of this material. The addition of the two plasticizers contributed to a better dispersion of the nanoparticles by increasing the interaction between the cellulosic reinforcements and the matrix, whereas the increased crystallinity of the incubated samples in a second stage in composting provoked a reduction in the disintegration rate.

## 1. Introduction

Biodegradable polymers have attracted great scientific and technological interest worldwide, due to their important role in the reduction of plastic waste problem. In this sense, several biodegradable polymers have been investigated during the past as possible replacement options to non-degradable polymers currently used in film production [1].

Poly(hydroxybutyrate) (PHB), a poly(hydroxyalcanoate) (PHA) synthesized by controlled bacterial fermentation [2], is a high crystalline polymeric material; however, one of its main drawbacks is the melting temperature close to the degradation temperature [3], responsible for a small processing window in the case of melt extrusion [4,5]. PHB melting temperature can be lowered, avoiding the thermal degradation of the polymer, by considering chemical or physical modifications [6], by introducing structural units into the PHB backbone or blending with other biopolymers, as in the case of PLA (polylactic acid)/PHB blends [4]. Other than these possibilities, the application of biopolymers can be extended by considering the addition of modifiers/plasticizers, by copolymerization or blending. Some applications of these materials are as packaging, such as for fast food, hygiene, single-use cups, and agricultural foils [6,7].

The use of plasticizers, for example, could represent an approach to overcome the disadvantages of PHB and to improve the mechanical characteristics, such as impact strength, deformability, and ductility. A requirement for PHB plasticizers should be their biodegradability: citrate-based plasticizers, derived from naturally-occurring citric acid, have already been successfully considered. Acetyl tributyl citrate (ATBC) is considered one of the best selections for polymer plasticization in biomedical applications, and it is known to bring, for example, improved thermal stability when added to the PHAs and their blends [8]. The literature also reports the use of plasticizers that are not only cheap, but also readily available on the market, such as dodecanol, lauric acid, tributyrin, and trilaurin. In this scenario, the work of Puglia and co-authors reported the effect of tributyrin on processing conditions and final properties of organically-modified clay-based poly(3-hydroxybutyrate) nanocomposites [9]. The authors observed that intercalated nanocomposites were obtained by adding tributyrin as a plasticizer and modified clays as reinforcement phases, and proved the positive effect of the plasticizer in tuning the disintegrability in composting conditions of the produced materials. The presence of the plasticizer, in fact, induces a modification in the diffusion mechanism, consequently enhancing the degradation in compost [9].

Alternatively, the use of natural reinforcement phases, and so a composite approach, to improve specific properties of biopolymers, represents a promising method. For a long time, natural lignocellulosic materials with their main constituents (cellulose, hemicelluloses, and lignin) have been used by a number of industries in forest products, paper, textiles, etc. [10,11]. The second generation of micro- and nano-scaled cellulose-based materials, is represented by micro- and nano-fibrillated cellulose nanofibers (MFC and NFC, respectively), nanocrystalline cellulose, or bacterial cellulose (BC).

Nanocrystalline cellulose (NCC), which can be obtained in two different forms (cellulose nanocrystals (CNCs) and nanowhiskers (CNWs)), has a typical rod-shaped morphology, with dimensions below 100 nm in diameter and tens to hundreds of nanometers in length. Its characteristics strongly depend on species, cultivar, and agronomical factors of the raw material. It is even proved that the yield of the extraction process depends on the procedure selected for the extraction of the crystalline phase. CNC extraction can be performed by using enzymatic or chemical treatments, with the aim of removing amorphous content from plant sources and leave highly-crystalline cellulose. One of the most common extraction methods considered a combination of chemical pretreatments suitable for the removal of the lignin, followed by an acidic treatment, usually performed by considering sulfuric acid, that obtains cellulose nanocrystals in an aqueous suspension [12,13]. CNCs have been successfully incorporated into PLA [14,15,16], PHB matrices [17,18], or PLA/PHB-based blends [19], showing an enhancement in the mechanical properties better than other reinforcing materials, such as nanoclays or carbon-based materials. Bacterial cellulose (BC) or bacterial nanocellulose (BNC) is formed by acetic acid aerobic bacteria, and it has, in comparison to cellulose obtained from plant sources, the advantage of being free of secondary components (lignin, hemicellulose, and pectin). According to this, BC usually consists of high molecular weight and high crystallinity material with high mechanical performance [20] that favors use in the production of composite materials. However, these remarkable properties of BC are hindered by its extremely hydrophilic behavior, which is responsible for BC’s poor interfacial adhesion to hydrophobic polymers [21]. 

The relationship between structure and properties of nanocomposites based on PHB reinforced with CNCs and BC was studied in a previous work [22]. Nanocomposites were produced by casting, using *N*,*N*-dimethylformamide (DMF) as the solvent. The use of this aprotic and polar solvent allows using some hydrophobic matrices [23]. These nanocomposites that were obtained with low percentages of CNC (2 to 6 wt %) have shown improved thermal stability, strength, and stiffness. Additionally, the barrier properties and transparency of PHB were maintained and, in some cases, enhanced due to the good dispersion and high interaction between CNC and the matrix. In addition, PHB/BC nanocomposites presented less dispersion degree and weak barrier capacity with respect to BC reinforcement, because of its higher aspect ratio on the order of microns and low interfacial adhesion.

In the present work, PHB-based formulations, reinforced with either BC or CNCs and plasticized using a molecular plasticizer (tributyrin) or a polymeric plasticizer (poly(adipate diethylene)), were produced by solvent casting. Their morphological, thermal, wettability, and chemical properties were investigated. Furthermore, the effect of both plasticizers (added at 20 wt % with respect to the PHB content) and bio-based selected nanofillers added in different amounts (2 and 4 wt %), when added to plasticized films, were investigated in terms of the disintegrability rate in simulated composting conditions. 

## 2. Materials and Methods 

### 2.1. Materials

Poly(3-hydroxybutyrate) (PHB) (*M*w = 600,000 g/mol) was kindly supplied by Biocycle^®^. (PHB Industrial Brasil S.A, Serrana, Brazil) Microcrystalline cellulose (MCC, dimensions of 10–15 µm) was supplied by Sigma-Aldrich^®^ (St. Louis, MO, USA). The plasticizers, tributyrin (TB) (*M* = 302 g/mol) and poly(adipate diethylene) (A) (*M*n = 2500 g/mol), were also supplied by Sigma-Aldrich^®^ (St. Louis, MO, USA). *N*,*N*-Dimethylformamide (DMF) and sulfuric acid were from Cicarelli^®^ (Laboratorios Cicarelli, San Lorenzo, Santa Fe, Argentina).

### 2.2. Methods

#### 2.2.1. Synthesis of Cellulosic Nanostructures

Cellulose nanocrystals (CNC) were obtained by hydrolysis from microcrystalline cellulose (MCC) in a sulfuric acid solution 64% *w*/*w* at 45 °C for 40 min following the recipe used by [24]. Subsequently, the suspension was cooled in an ice bath and diluted with bi-distilled water to quench the reaction. Then, the suspension was dialyzed against water to neutral pH. Ultrasonic sonication (tip sonicator, Cole Parmer, Chicago, IL, USA) was performed four times, for a total time of 20 min. The resultant cellulose nanocrystal aqueous suspension was approximately 1% (*w*/*w*) by weight and the extraction yield was ca. 10%. Finally, the synthesized nanocrystals were lyophilized by a VirTis benchtop SLC lyophilizer (SP Scientific, SP Industries, Warminster, PA, USA). Bacterial cellulose (BC) was produced by *Gluconacetobacter xylinus* by using alternative low-cost carbon sources (glycerol from biodiesel production and grape bagasse) [25]. The morphology of extracted cellulose nanocrystals and synthesized bacterial cellulose was investigated by means of field emission scanning electron microscopy (FESEM, Supra 25-Zeiss, Carl Zeiss AG, Oberkochen, Germany). 

**Figure 1:**
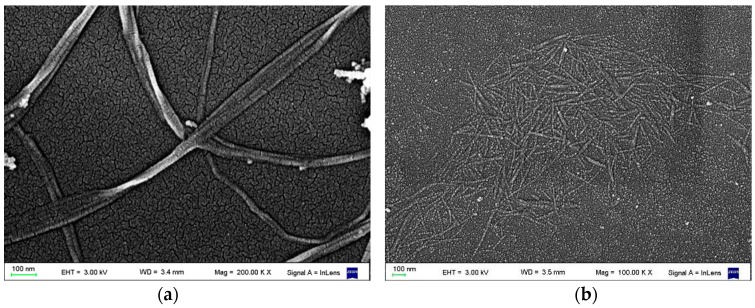
FESEM images of (**a**) CNC and (**b**) BC.

The obtained CNC showed the typical acicular structure with the dimensions ranging from 130–290 nm in length and 3–6 nm in width (Figure 1a). BC showed a ribbon-like shape several micrometers long and a rectangular cross-section with width and thickness in the range of 35–70 and 13–24 nm, respectively (Figure 1b).

#### 2.2.2. Preparation of PHB Based Nanocomposites

PHB based systems were produced by a solvent casting procedure. In all cases, PHB pellets were dissolved in DMF by stirring at 116 °C, avoiding thermal degradation of PHB. Nanocomposites were obtained using suspensions in DMF with different amounts of CNC (2 and 4 wt %) after being sonicated during 30 min, or BC (2 wt %) after being homogenized two times for 15 min for each one. Each suspension was added to PHB solution and then the mixtures were again sonicated or homogenized for 10 min, depending on the nanofiller. Homogenization was carried out with an IKA^®^ T18 basic Ultra Turrax (IKA^®^ Works, Inc., Wilmington, NC, USA).

USA. Plasticized PHB samples were prepared by adding 20 wt % of each plasticizer, TB or A, to PHB solution and, in the case of plasticized nanocomposites, sonicated CNC or homogenized BC suspensions were incorporated into the mix of PHB and plasticizer and then they were again sonicated or homogenized, also depending on the nanofiller, for 10 min. The solutions were then poured into Petri dishes and kept in the oven at 80 °C for 12 h to allow solvent evaporation. The materials were kept at ambient temperature for 15 days, in order to complete the crystallization of PHB. The nomenclature used to identify each produced sample is described in Table 1. 

#### 2.2.3. Characterization Methods

Fourier transform infrared (FTIR) spectra were acquired with a Mattson Genesis II spectrometer (Mattson Instruments, Madison, WI, USA), by considering a spectral width of 400–4000 cm^−1^, 32 accumulations, and a resolution of 4 cm^−1^. Five samples of each material were analyzed. Obtained spectra were normalized to the intensity of the band at 2933 cm^−1^, which corresponds to the group CH_2_ that is present in all samples.

Thermal degradation measurements were performed in a TA instruments Auto-MTGA Q500 Hi-Res thermogravimetric analyzer (TGA) (TA Instruments, New Castle, DE, USA). Temperature was raised from room temperature up to 700 °C using 10 °C/min as heating rate. Three analyses were performed for each sample. All runs were carried out under a nitrogen atmosphere (30 mL/min) in order to prevent any thermo-oxidative reaction. 

The calorimetry analysis (DSC) was carried out by using a Perkin Elmer DSC instrument (Perkin Elmer, Waltham, MA, USA). Five samples for each material were heated from room temperature to 200 °C at a heating rate of 10 °C/min. PHB crystallinity degree was determined using Equation (1), considering the area of the melting peaks as the melting enthalpy:

(1) $$Xc_{PHB}=\frac{\Delta H_{m}}{\Delta H_{m}^{0}\cdot w_{PHB}}\times 100$$

where $\Delta H_{m}$ is the measured value for PHB melting enthalpy, $\Delta H_{m}^{0}$ is the melting heat associated with pure crystalline PHB (146 J/mol) [26], and *w_PHB_* is the weight fraction of PHB in the composite.

Water contact angle was evaluated by using the sessile drop method. Contact angles of the drops were measured by means of a MV-50 camera, 6× zoom, and images were taken by using NIH imaging software. The measurement values represent the mean value of 10 drops.

X-ray Diffraction (XRD) measurements were performed with KCuα (k = 1.54 Å) radiation in a Philips PW 1710 X-ray diffractometer system (PANalytical, Almelo, The Netherlands) (instrument parameters: 40 kV and 40 mA, at 2°/min in the 2 h range from 5° to 60°).

The microstructure of the film surfaces was detected by using a field emission scanning electron microscope (FESEM), Supra 25-Zeiss, Carl Zeiss AG, Oberkochen, Germany. Specimens were fractured by immersion in liquid nitrogen, gold-coated, and observed by using an accelerating voltage of 4 kV.

#### 2.2.4. Disintegrability in Composting Conditions 

Disintegrability in composting conditions was carried out according to the standard ISO 20200. The test regulates, at the laboratory-scale, the degree of disintegration (D) of plastic materials under simulated aerobic composting condition at 58 °C and 50% of humidity. PHB and PHB binary and ternary film formulations of dimensions 15 mm × 15 mm × 0.03 mm were weighed and buried into the organic substrate at 4–6 cm depth in perforated boxes. The aerobic conditions were guaranteed by mixing the soil while the 50% of humidity was guaranteed regenerating the water content into the compost boxes during the test. *D* was calculated by normalizing the sample weight at different days of incubation respect to the initial value, by using Equation (2):

(2) $$D=\frac{m_{i}-m_{r}}{m_{i}}*100$$

where *m_i_* = is the initial weight of the dry plastic mass; *m_r_* = is the weight of the dry plastic material after the test. 

In order to simulate the compost soil, a solid synthetic waste was prepared, mixing sawdust, compost inoculum, starch, rabbit food, sugar, oil, and urea. The samples tested were removed at different times (7, 14, 21, and 28 days), washed with distilled water, and dried in an oven at 37 °C for 24 h. Photographs of all produced and tested formulations were taken for visual comparison to evaluate the modifications in terms of color and the dimension variations during the different days in the soil. Finally, the surface microstructure of PHB and PHB composites at 14 days of incubation in composting was investigated by FESEM (Supra 25-Zeiss, Carl Zeiss AG, Oberkochen, Germany), after gold sputtering. XRD and FESEM were performed with a piece of each sample with 14 days under compost conditions. 

## 3. Results

### 3.1. Characterization of PHB-Based Nanocomposites

**Figure 2:**
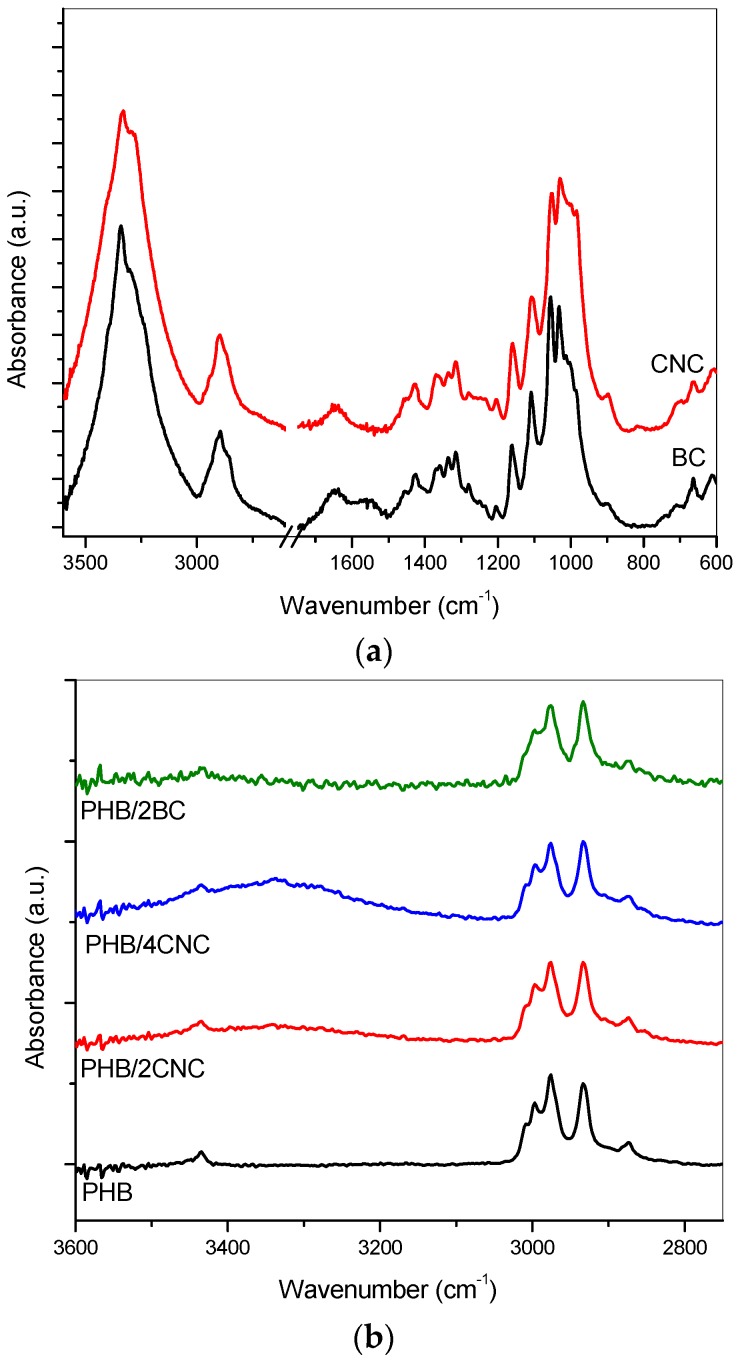
FTIR spectra of CNC and BC (**a**), PHB and PHB nanocomposites with different CNC and BC loading (**b**).

Chemical structure of PHB nanocomposites materials before degradation in compost was studied by FTIR. As observed in our previous work [22], characteristic PHB bands were not significantly modified by the addition of cellulose-based nanoparticles. As reported in the cited reference, the spectral band located at 2900 cm^−1^ is assigned to C–H stretching vibrations, while the spectral bands located at 1420, 1375, 1335, and 1278 cm^−1^ correspond to the in-plane bending of the former C–H groups. Other peaks located at 900 cm^−1^ and 1160 cm^−1^ are assigned to C–O–C stretching of glycosidic bonds. The presence of CNC (Figure 2a) was noticed by the absorbance increase at 3350 cm^−1^, attributed to the –OH group, even at the low amount of 2 wt % (PHB/2CNC) (Figure 2b). Although this peak is characteristic of both fillers structure, the BC nanocomposite (PHB/2BC) showed a broadening of this band, indicating a possible overlapping stretching with the PHB signal or the reorganization of hydrogen-bound –OH.

**Figure 3:**
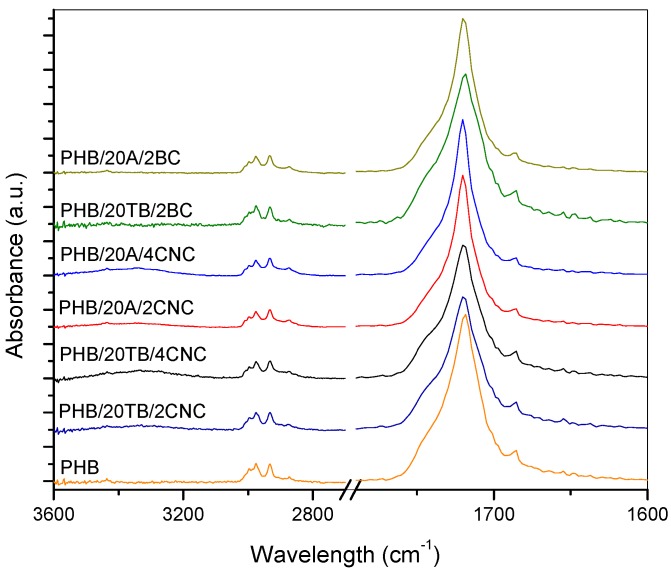
FTIR spectra of PHB ternary composites.

FTIR spectra of ternary composites containing both plasticizers and CNC addition also showed an increase of the absorbance corresponding to –OH group (Figure 3). Furthermore, typical PHB bands of ester carbonyl groups at 1740 cm^−1^ (characteristic of the amorphous state) and at 1720 cm^−1^ (typical of the crystalline state) were observed in the spectra [27].

These bands became broader due to the hydrogen bond interactions between hydroxyl groups of nanocellulose and PHB and TB carbonyl groups, as proposed by Arrieta et al. [28]. These results indicate that only CNC composites present superficial –OH groups and this promotes the hydrophilic character of the films.

**Figure 4:**
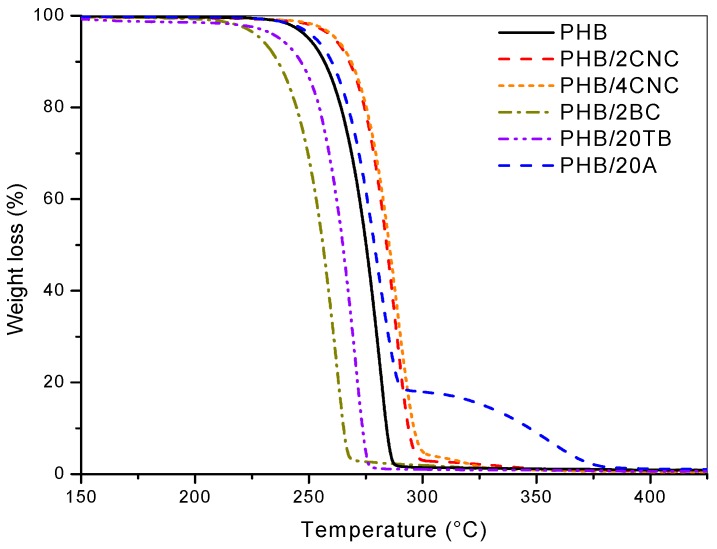
TGA curves of PHB and PHB binary samples.

**Figure 5:**
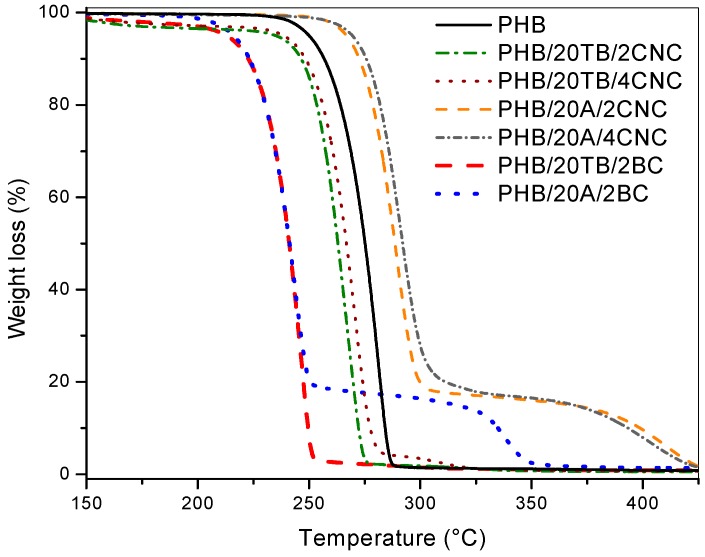
TGA curves of PHB and PHB ternary nanocomposites.

TGA analysis of produced materials before simulated compost degradation was performed and weight loss curves of binary and ternary samples are presented in Figure 4 and Figure 5, respectively. Nanocomposites with CNC as filler presented higher thermal stability when compared to neat PHB. On the other hand, thermal stability of BC nanocomposites was reduced, confirming the supposed interactions between matrix and CNC [29]. Additionally, in a previous work [18], it was reported that hydrogen interactions between C=O groups in PHB and –OH groups in CNC could be detected in related FTIR spectra. Instead, BC presents poor interfacial adhesion with PHB due to its low compatibility, related to its higher aspect ratio that hinders the contact between both constituents [22,30]. Thermal degradation of plasticized PHB samples showed two drops corresponding to the degradation steps of both plasticizer and matrix (Figure 4). 

In the case of TB-containing samples, degradation started at lower temperatures than PHB, due to TB vaporization [31] and it was observed that the maximum degradation temperature of PHB was reduced by the addition of TB. This could be due to enhanced PHB chain mobility, in agreement with the plasticizing effect of TB and the incorporation of ester groups presented in the TB chemical structure, which promotes catalytic degradation reactions [32], reducing matrix thermal stability. The weight loss curve of PHB plasticized with A presented a first drop attributed to matrix degradation and a second drop due to A evaporation. It was found, in this case, that the matrix thermal stability was enhanced, attributing this behavior to the higher thermal stability of A plasticizer with respect to neat PHB [33]. 

Thermal stability of ternary nanocomposites could be even explained by additive or filler effects (Figure 5). Ternary samples presented two drops corresponding to the degradation of the additive and the matrix. As already observed, thermal stability of ternary nanocomposites was compromised by the addition of TB, while increased in the case of A plasticizer [34]. The plasticizer presence decreased the onset degradation temperature, in good agreement with other PHB-plasticized materials [8]. BC also produced a marked detrimental effect on thermal performance. On the other hand, the CNC addition improved the thermal stability of the nanocomposites, moving the onset degradation temperature of the systems to higher values. In particular, plasticized ternary nanocomposites containing A presented higher stability with respect to neat PHB, due to higher thermal stability of A and CNC.

**Figure 6:**
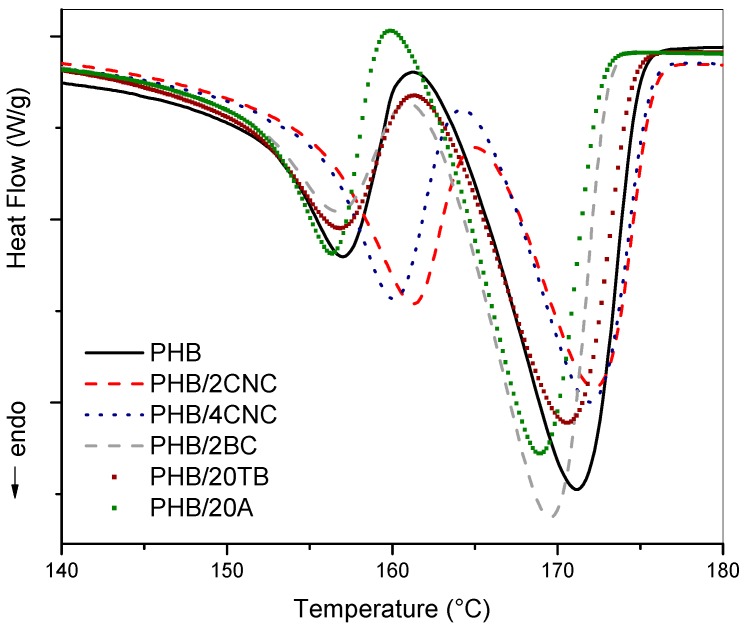
DSC curves of PHB and PHB binary samples.

In order to study the melting behavior of produced materials, DSC analysis was also performed. In Figure 6, melting behavior of binary blends is reported. Double-melting points of PHB were observed for all samples, demonstrating a melting-recrystallization-remelting process. Values for the melting temperature of original crystals (*T*_m1_) and PHB crystallinity (*X*_c PHB_) of the nanocomposite samples are detailed in Table 2. 

No significant influence on melting transition was observed with the addition of neither plasticizers nor BC. On the other hand, nanocomposites with CNC presented an increment of Tm_1_, due to the formation of higher lamellar thickness or greater crystalline order [35], in agreement with TGA results [22]. It was observed that melting behavior did not present substantial differences between these samples. Nevertheless, their crystallinity degree was slightly increased, especially in the case of samples formulated with TB.

**Figure 7:**
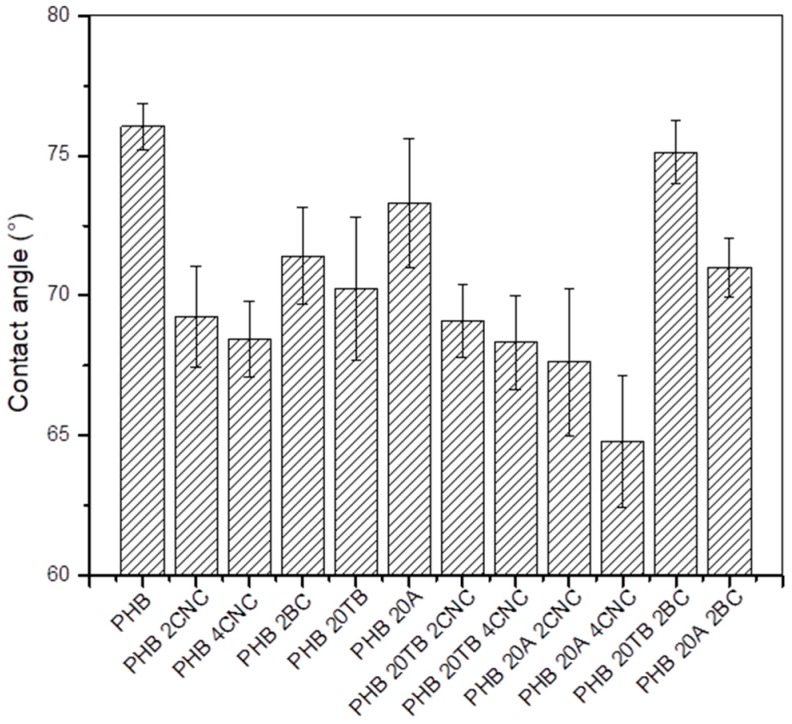
Contact angles of PHB nanocomposites and plasticized samples.

Contact angle was analyzed to compare surface wettability of binary and ternary composites and the results are presented in Figure 7. Neat PHB is a hydrophobic polymer and, according to this, it presented an angle of (76 ± 1)°. 

CNC addition reduced the hydrophobic character of the final material, probably due to the incorporation of –OH group on surface of the films, as previously observed by FTIR characterization. BC addition also reduced the water contact angle and it could be due to a modified diffusion process [29], conferred by poor dispersion of this nanofiller [22]. Plasticizers also reduced the contact angle value, since they favor polymer chain mobility and, consequently, diffusion through the films. In general, studied formulations presented a contact angle greater than 65° (only the ternary sample with A and 4 wt % of CNC showed an angle around 65°), indicating a low water affinity to the material’s surface that benefits their potential use in food packaging applications [36].

### 3.2. Disintegrability in Composting Conditions

**Figure 8:**
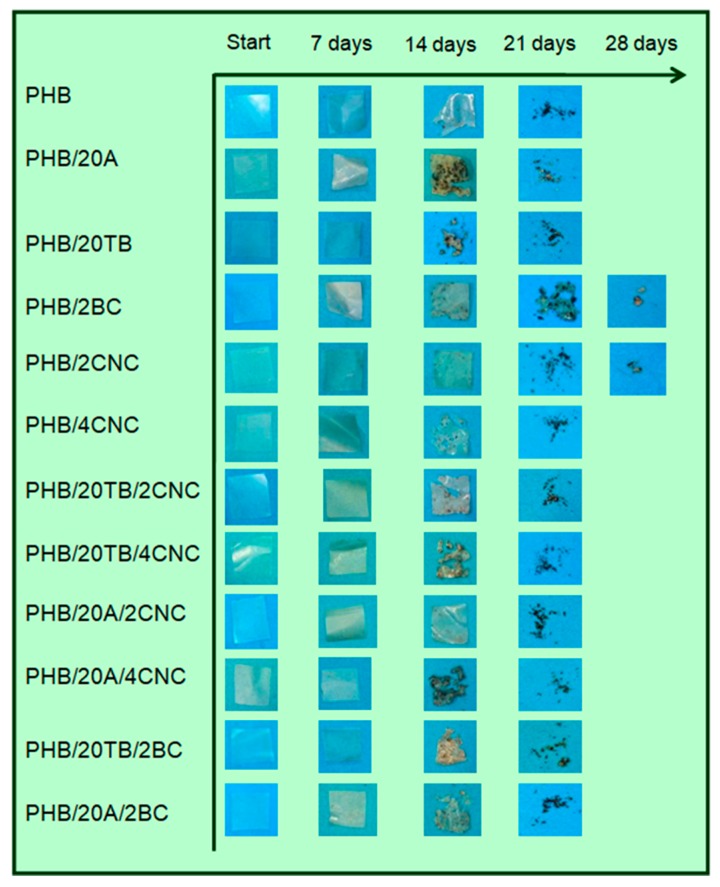
Visual appearance of PHB nanocomposites at different incubation times under composting conditions.

The visual appearance of degraded films at different time of disintegration in composting conditions is shown in Figure 8. The performed study confirms the biodegradable character of all formulations, reaching the goal of 90% of disintegrability according to ISO 20200 in about 21 to 28 days, depending on the film’s composition. During the first week, opacity and deformation of the surface was detected for all materials, whereas during the second week sample erosion was observed. Changes in the film’s color at the first stages of the test were related to the hydrolytic degradation process [37]. At higher testing times, samples broke into small pieces and irregular surfaces, even with apparent color changes related to the degradation stage.

**Figure 9:**
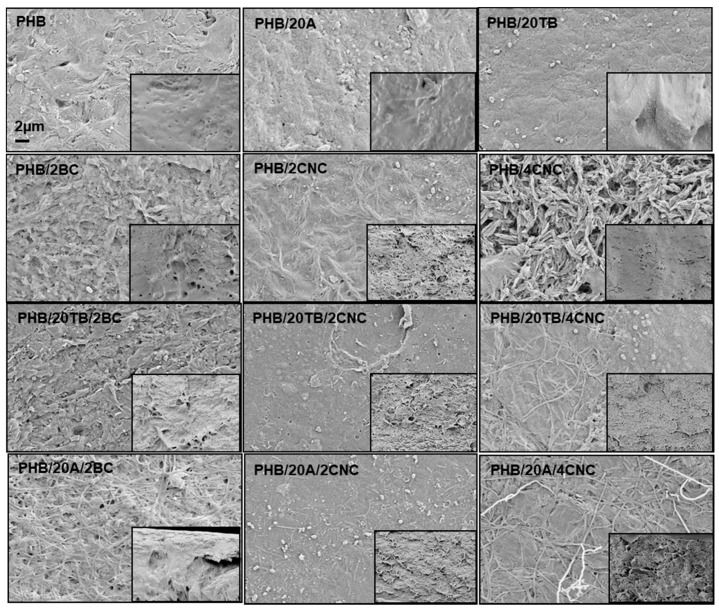
FESEM images of disintegrated samples after 14 days under composting conditions.

In addition, Figure 9 shows FESEM images of the samples after 14 days in compost (at time 0 in the inset) with evident signs of surface erosion. Samples with 4 wt % of CNC evidenced the progressive degradation, showing pores permitting the easy access of microorganisms to the polymer bulk. A clear increase of the disintegration phenomenon was observed in PHB films due to the addition of both plasticizers: this results followed the tendency already observed in the case of surface water contact angle measurements (higher values of water contact angle in the case of A containing films with respect of TB plasticized PHB), since the polymer disintegration starts by the hydrolysis process. Even if wettability is strongly affected by the surface chemical and topographical properties, water diffusion through the polymer matrix is strictly related to the crystallinity of the system: PHB/20A samples containing CNC or BC showed the lowest crystallinity values caused by the synergic effect of PHB, CNC/BC, and A on the crystallization of PHB (as already commented in Table 2) and the amorphous regions are less resistant to hydrolysis and degradation processes. 

**Figure 10:**
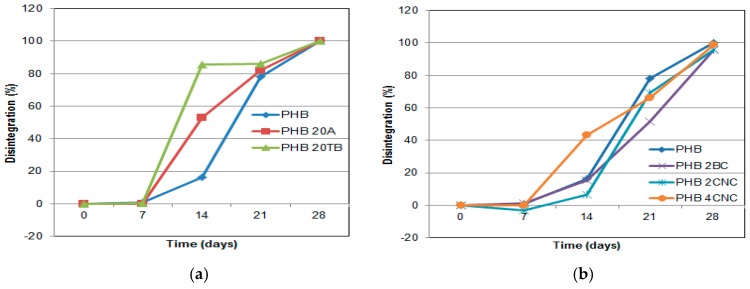
Disintegrability of plasticized PHB- (**a**) and cellulose-based nanocomposites (**b**).

Figure 10 reports the evolution of disintegrability during composting time of binary films. A clear increase in the disintegrability rate was observed for plasticized PHB samples at 14 days in compost (Figure 10a). It was verified that PHB suffers surface erosion during the first stage of composting [38] instead of bulk erosion. The inclusion of plasticizers modified the diffusion pathway and accelerated the bulk degradation [31]. Moreover, the inclusion of ester bonds presented in the TB structure could speed up disintegrability because they are susceptible to PHB depolymerases. The addition of CNC is able to retard the degradation rate for samples with 2 wt % of CNC and accelerate it for higher percentages (Figure 10b). In the case of BC-based binary nanocomposite, disintegrability evolution resulted similar to PHB behavior. In contrast to BC, CNC inhibits water diffusion due to strong interactions between the matrix and filler and contributes to slow down the microorganism attack at lower CNC percentages [22,39].

**Figure 11:**
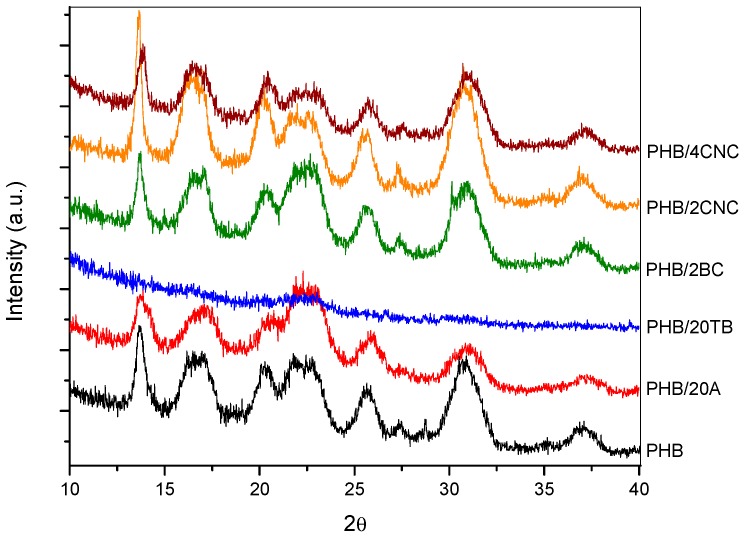
XRD spectra of PHB and binary samples with 14 days under compost conditions.

Abe et al. [40] found that the degradation rate decreases with an increase in lamellar thickness and this could be another reason that justifies the disintegrability reduction of PHB containing 2 wt % of CNC [31], as already observed by DSC for the different samples prior to degradation. Otherwise, the addition of hydrophilic filler contributes to accelerate the degradation rate, and this could be the reason for the accelerating effect due to the increased filler percentage. This effect was also identified by Bitinis et al. [39], but it was counterbalanced with the opposite effect of diffusion reduction [22]. Enzymatic degradability decreases with an increase in crystallinity [41]. Additionally, water and chemical diffusion are highly affected by the crystallinity of the system. Although all samples presented similar PHB crystallinity (Table 2), the increase of crystallinity during degradation should be taken into account, since disintegration test was carried out at 58 °C. At this temperature, PHB chains have enough energy to induce crystallization. In particular, crystallization is assisted in nanocomposites due to nucleating effects of the cellulosic filler [42,43,44,45]. Crystallinity changes during degradation could be the reason of the disintegrability rate delay observed for nanocomposites after 21 days under compost conditions. In order to confirm the assumption, crystallinity changes during degradation for the binary formulations were studied by XRD analyses. Figure 11 presents the XRD spectra of PHB and binary samples after 14 days buried in soil. The crystalline degree of all samples was improved due to the reduction of the spectrum area attributed to the amorphous halo, proving that the amorphous phase is more susceptible to be hydrolyzed than the crystalline phase. In addition, a certain increase of crystalline peaks’ intensities for the samples with 2 wt % of CNC and 2 wt % of BC was observed, sustaining that crystallization of nanocomposites could be induced during incubation. A reduction of the plasticized samples’ peaks was also observed, mainly those of the sample with TB, in accordance with the higher decomposition of these samples.

**Figure 12:**
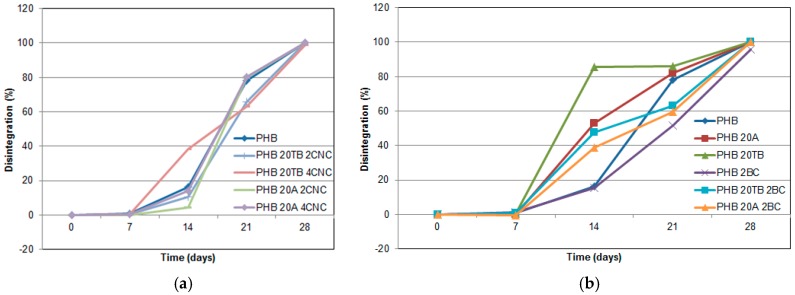
Disintegrability of CNC- (**a**) and BC-based (**b**) plasticized nanocomposites.

Disintegrability curves of ternary nanocomposites loaded with both plasticizers and CNC are shown in Figure 12a. It was observed that PHB/TB/CNC composites presented disintegrability patterns similar to binary films with CNC, due to the nanoparticle effect that predominates the disintegration rate of plasticized films. Otherwise, ternary nanocomposites PHB/A/CNC presented an evolution in compost similar to PHB showing, at a first stage, a reduced disintegrability rate due to the presence of CNC. However, the sample PHB/20A/4CNC has a visual appearance at 14 days in soil that could indicate advanced degradation (Figure 8), similarly to PHB/4CNC or PHB/20TB/4CNC. The slight variation on disintegration percentage could be explained considering that wettability favors liquid diffusion through the film (Figure 7), leading to a high content of absorbed water. In Figure 12b, disintegrability evolution of binary and ternary nanocomposites with both plasticizers and BC is presented. 

**Figure 13:**
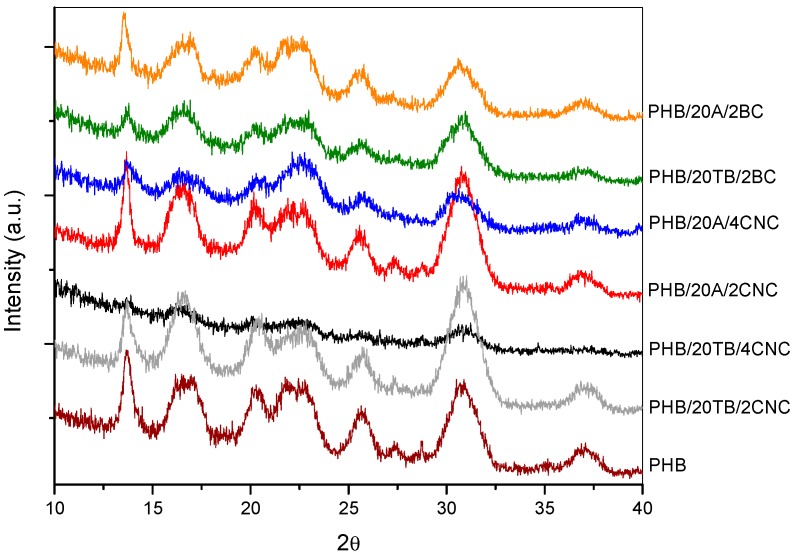
XRD spectra of PHB and plasticized nanocomposites with 14 days under compost conditions.

It was observed that ternary composites with BC presented an accelerated degradation rate at the first stage in compost when compared to PHB, probably due to the degradation of plasticizers. Then, a delay in the disintegrability rate occurs, similarly to nanocomposites with CNC, due to an increased crystallinity of degraded samples. Figure 13 shows the XRD spectra of PHB and ternary samples with 14 days buried in soil. Once more, the crystalline degree of all samples was altered, confirming that disintegration rate decreased with increasing crystallinity values even in the case of BC-containing samples. Moreover, the spectrum of the ternary composite with TB and 2 wt % of CNC presented an increment of peaks intensity, as already observed for the binary sample with 2 wt % of CNC (Figure 11). 

## 4. Conclusions

Disintegration in composting conditions of plasticized PHB nanocomposite films containing cellulosic-based nanoreinforcements was tested. The plasticized PHB films exhibited a marked acceleration in disintegration, since the addition of the plasticizer increases the wettability of the material and diffusion through the matrix, due to the greater mobility of the polymer chains. Consequently, water is fed in the first stage of composting, then the diffusion of small molecules produced during disintegration, from the inside of the material to the outside, occurred. In addition, it was observed that, particularly, TB can contribute to the increase in the rate of disintegration by adding a high amount of ester groups, susceptible to the attack of depolymerase enzymes of PHB.

In the case of binary nanocomposites, it was observed that in a first stage in composting, the CNC aggregate slows down the rate of degradation in samples with low CNC content (2 wt %) and accelerates it, increasing the percentage. The sample with 2 wt % of CNC has a lower permeability to water vapor than the PHB and, therefore, the diffusion of the water to the sample core, as well as degradation products to the outside, is restricted. This aspect may contribute to delaying the attack of microorganisms against the material. On the other hand, the addition of a higher CNC content can increase the rate of degradation as it can absorb more water content due to its hydrophilic character, which favors the hydrolysis of the material. In contrast, nanocomposites with BC presented rather comparable disintegrability behavior in composting with that of PHB, possibly due to the lower hydrophilic character of this material in relation to nanocomposites with CNC.

The study of plasticized PHB nanocomposites showed results that can be compared to the behavior of binary samples. The nanocomposites PHB/TB/CNC presented similar behavior as binary nanocomposites with CNC, since the addition of TB can contribute to the dispersion of the nanoparticles by increasing the interaction between CNC and the matrix. The nanocomposites with A presented a similar evolution to the PHB, possibly due to a higher water absorption that increases the total mass of the sample, hiding the disintegration, as a consequence of a high permeability. The compounds with plasticizer and BC exhibited a behavior similar to that of plasticized PHB without reinforcement (binary mixtures), due to the low dispersion of BC in the matrix. In addition, it was found that all the films, mainly the ones containing nanoparticles, present a reduction in the rate of disintegration in a second stage in composting, due to the increased crystallinity of the incubated samples. It was corroborated that the incubated samples present an important reduction of the amorphous phase, since this one is more susceptible to be hydrolyzed than the crystalline one.

## Figures and Tables

**Table 1:** Nomenclature used for each sample.

Materials	Compositions
PHB	Neat PHB
PHB/2CNC	PHB/2 wt % CNC
PHB/4CNC	PHB/4 wt % CNC
PHB/2BC	PHB/2 wt % BC
PHB/20TB	PHB/20 wt % TB
PHB/20A	PHB/20 wt % A
PHB/20TB/2CNC	PHB/20 wt % TB/2 wt % CNC
PHB/20TB/4CNC	PHB/20 wt % TB/4 wt % CNC
PHB/20A/2CNC	PHB/20 wt % A/2 wt % CNC
PHB/20A/4CNC	PHB/20 wt % A/4 wt % CNC
PHB/20TB/2BC	PHB/20 wt % TB/2 wt % BC
PHB/20A/2BC	PHB/20 wt % A/2 wt % BC

**Table 2:** Melting temperature (*T*_m_)/crystallinity (*X*_c PHB_) of PHB nanocomposites/plasticized samples.

Materials	*T*_m1_ (°C)	*T*_m2_ (°C)	*X*_c PHB_ (%)
PHB	157.0	171.0	55.4
PHB/2CNC	160.0	171.4	55.9
PHB/4CNC	159.5	171.2	55.4
PHB/2BC	157.8	170.9	55.5
PHB/20TB	156.7	170.7	57.0
PHB/20A	156.7	169.4	53.4
PHB/20TB/2CNC	154.5	169.2	62.7
PHB/20TB/4CNC	154.7	169.6	59.8
PHB/20A/2CNC	155.3	169.4	57.1
PHB/20A/4CNC	154.1	168.9	56.8
PHB/20TB/2BC	155.6	169.5	60.7
PHB/20A/2BC	155.4	169.0	53.5
